# Silencing circular RNAPTPN12 promoted the growth of keloid fibroblasts by activating Wnt signaling pathway via targeting microRNA-21-5p

**DOI:** 10.1080/21655979.2022.2029108

**Published:** 2022-01-22

**Authors:** Fei Liu, Tao Li, Xiaoan Zhan

**Affiliations:** a Department of Dermatology, Jinhua People’s Hospital, Jinhua, Zhejiang, China; b Department of Dermatology, Cancer Hospital Affiliated to the University of Chinese Academy of Sciences, Hangzhou, Zhejiang, China; c Oncology Surgery, Zhejiang Jinhua Guangfu Tumor Hospital, Jinhua, Zhejiang, China

**Keywords:** circPTPN12, miR-21-5p, keloid, keloid fibroblasts, cell viability

## Abstract

Keloid is a skin disease marked by fibroplasia, and fibroblasts viability plays a considerable part in keloid. Our research was devoted to assessing the involvement and mechanism of circPTPN12 in keloid. The level of circPTPN12 and miR-21-5p was estimated by qRT-PCR in keloid tissues and cells. MTT analysis was devoted to evaluating the multiplication of keloid fibroblasts. Additionally, transwell assay was dedicated to verifying cell migration and invasion. Furthermore, keloid fibroblasts apoptosis level was assessed adopting flow cytometry, and the relevancy between miR-21-5p and circPTPN12, miR-21-5p, and SMAD7 was assessed by dual luciferase assay. Similarly, RIP and RNA pull-down assay verified the relevance between genes. Moreover, levels of SMAD7 and proteins concerned in Wnt signaling pathway were appraised by Western blot. The level of circPTPN12 declined in keloid. circPTPN12 knockout could enhance the multiplication, migration, invasion, and decline apoptosis of keloid fibroblasts. Indeed, miR-21-5p could be packed with circPTPN12 sponge, SMAD7 was downstream effect factor of miR-21-5p, and miR-21-5p inhibitors partially reversed the promoting effect of silencing circPTPN12 on keloid formation. Otherwise, the level of SMAD7 was adjusted by circPTPN12 and miR-21-5p. Silencing circPTPN12 targeted miR-21-5p and activated Wnt pathway to accelerate keloid fibroblasts growth. Taken together, silencing circPTPN12 promotes the growth of keloid fibroblasts by activating Wnt pathway targeting miR-21-5p. CircPTPN12 may play a considerable part in keloid formation, which supplies a reference for molecularly targeted therapy keloid.

## Graphical abstract

**Figure:**
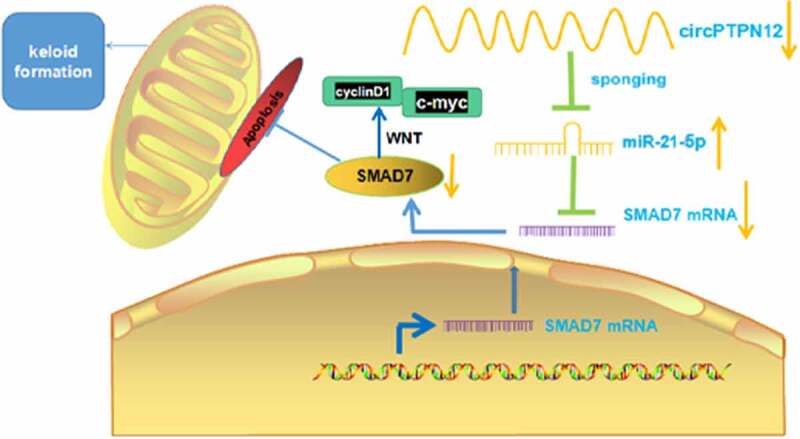

## Introduction

Keloid, also known as a pathological scar, is caused by the abnormal accumulation of collagen after a personal skin injuries mainly related to the excessive proliferation of fibroblasts and the deposition of extracellular matrix. Keloid is characterized by scabs and plaques on the surface of the skin. Whose color is bright red or lilac, with itching and tingling [1,2]. The investigation has validated that keloid formation involves various complex factors, such as cytokines, inflammatory factors, and gene regulation [3]. Keloid is supposed to be a benign skin tumor; although it is not malignant, it has an eminent recurrence in clinical [4,5]. In recent years, the formation and regulation mechanism of keloid has become a research hotspot in biomedicine, and keloid treatment needs to be broken through.

Previous researches have verified that many genes and transduction channels are devoted to keloid formation, including circular RNAs (circRNAs) [6]. CircRNAs are non-coding RNA, a class of particular RNA.CircRNA that lacks 5’ and 3’ ends and forms a closed loop by covalent bonds is more stable than linear RNA [7]. Previous studies have confirmed that circRNA regulates the viability of keloid fibroblasts, thereby controlling the process of keloid, including circ_101238 and circNPLP1 [8,9]. Among these circRNAs, circular RNA PTPN12 (circPTPN12) is a new molecule. Studies have confirmed that circPTPN12 is related to endometrial fibrosis and can promote endometrial fibrosis by regulating the circPTPN12/miR-21-5p/∆Np63α pathway [10]. Thus, the effect of circPTPN12 on keloid and its mechanism has not been studied.

It has been widely confirmed that CircRNAs have a hand regulating downstream target genes by playing the function of microRNA (miRNA) sponge [11,12]. Studies have revealed that miR-21-5p is abnormally expressed in the occurrence of cancer [13], cardiovascular diseases [14], and nervous system disease [15]. Moreover, in keloids, autophagy and metastasis of keloid fibroblasts irradiated by electron beam inhibited miR-21-5p and effectively prevented local invasion and recurrence [16]. Similarly, Li et al have confirmed that markedly enhanced miR-21-5p in keloid epidermis, the migration, and invasion of keloid keratinocytes are concerned in miR-21-5p [17]. Nevertheless, the function of the circPTPN12/miR-21-5p axis in adjusting keloid fibroblast activity is not yet specific. SMAD family member 7 (SMAD7), a member of the SMAD family, has been validated to be referred to cancer progression as a cancer-promoting or suppressing factor [18,19]. Li et al have confirmed that exocrine SMAD7 secreted by keloid fibroblasts is significantly more than normal skin fibroblasts, and exocrine miR-21 promotes the multiplication of keloid fibroblasts inhibiting SMAD7 [20]. Additionally, SMAD7, as a downstream effector, affects the proceeding of lung cancer cells mediated by miR-21-5p [21]. Correspondingly, in keloid, the differentially expressed circRNAs are related to miRNA-mRNA [22]. However, the involvement of circPTPN12/miR-21-5p/SMAD7 in keloid has not been elucidated. Based on the above research, the present research was devoted to probing the function of circPTPN12 adjusting multiplication, apoptosis, migration, and invasion of keloid fibroblasts to provide a reference for target treatment of keloid in clinical practice.

## Materials and methods

### Tissue specimen

A total of 40 patients who received keloid treatment in Jinhua People’s Hospital from April 2019 to May 2020 were selected for the study. During the operation, keloids and a small amount of surrounding normal skin tissue were collected. All tissue specimens were frozen in liquid nitrogen for 30 min of the same size and stored in-80°C refrigerator. Diagnosis of keloid was confirmed by the clinicians heading the recruitment teams. All patients who voluntarily participated in this study have signed an informed consent form. The Ethics Committee of our hospital sustained the research plan.

### Cell culture and transfection

Keloid fibroblasts and normal fibroblasts were separated from keloid tissues and normal tissues. Thenfostered in DMEM cell medium (Zeye Biotechnology, Shanghai, China), embodying 10% fetal bovine serum at 37°C, 5%CO_2_as described previously [23]. The fibroblasts were seeded in 6-well plates at a density of 2 × 10^5^/well and incubated to 40 ~ 50% confluence. Logarithmic growth phase cells were harvested for research and synthesis of siRNA negative control for circPTPN12, siRNA for circPTPN12, siRNA negative control for SMAD7, siRNA for SMAD7, miR-21-5p mimics, miR-21-5p inhibitor, and miR negative control (RiboBio, Guangzhou, China). According to the vendor manual, Lipofectamine®2000 (Hengfei Biotechnology, Shanghai, China) was used for cell transfection as described previously [24]. Cells at passages 3–5 were used for this study. Cells were divided into the normal group (NC), silencing circPTPN12(si-circPTPN12), siRNA control group (si-NC), The grouping of miR-21-5p, and SMAD7 is the same as above.

### Quantitative real-time PCR (qRT-PCR)

According to TRIzol(Invitrogen) steps, total RNA was extracted from keloid tissues and fibroblasts. Total RNA 450ng was reverse turned into cDNA; cDNA was employed as a template for PCR amplification 20 μL reaction system was used as described [25].The following primers were used, circPTPN12 F: 5ʹ-TCAAAATGAATCTCGTAGGCTGT-3ʹ and R 5ʹ-GTGCAAACGTTATGGGGTCT-3ʹ. miR-21-5p F: 5ʹ-ACACTCCAGCTGGGTAGCTTATCAGACTGA-3ʹ and R: 5ʹ-CTCAACTGGTGTCGTGGAGTCGGCAATTCAGTTGAGTCAACATC-3ʹ. SMAD7 mRNA F:5ʹ-ATGCTGTGCCTTCCTCCGCTG-3ʹ and R: 5ʹ-CCACGCACCAGTGTGACCGA-3ʹ. GAPDH F: 5ʹ-GGAGCGAGATCCCTCCAAAAT-3ʹ and R: 5ʹ-GGCTGTTGTCATACTTCTCATGG-3ʹ. U6 F: 5ʹ-CTCGCTTCGGCAGCACA-3ʹ and R: 5ʹ-AACGCTTCACGAATTTGCGT-3ʹ. The PCR reactivation conditions were pre-denaturation at 95°C, 15s, 60°C, 20s, 45 cycles. The data obtained from the experiment were analyzed relatively quantitatively using 2^(-ΔΔCt)^. (∆CT = objective gene-GADPH, ∆∆ = ∆CT experiment-∆CT) [26].

### MTT assay

MTT assay was performed as described previously [27]. After transfected 24 h, 8 × 10^3^fibroblastsper well were cultivated in 96-well plates. Another blank control group was set up, and only a cell culture medium was added. After 12, 24, 48, and 72 h of culture, 5 mg/mL MTT 10 μL (Zeye Biotechnology) was supplied and hatched for 4 h. After absorbing the supernatant, dimethyl sulfoxide (DMSO) was added, and it was put in a constant temperature oscillation box and oscillated 10 min at 37°C. Each well’s optical density(OD) value was assessed at the 490 nm wavelength on the automatic enzyme labeling instrument.

### Flow cytometry

Apoptosis assay was performed as described [28].Aftertransfection24 h, fibroblasts were inoculated into a 6-well plate with a concentration of 3 × 10^5^ cells per well, precooled with 70% ethanol, and placed overnight at a temperature of 4°C. 10 μL Annexin V FITC and 5 μL propidium iodide staining solution (0.25 mg/mL) were added and mixed gently, then incubated 20 min at room temperature and avoided light was detected by flow cytometry.

### Transwell assay

The transwell tests were conducted as described [29]. The diluted Matrigel (3.9 μg/μL) 60 ~ 80 μL was added to the polycarbonate film in the upper chamber of Transwell, and the Matrigel was polymerized into gel at 37°C 30 min. In the lower chamber of Transwell, 10% fetal bovine serum (FBS) 600 μL was added as chemokine; the serum-free medium of the cells to be tested was made into single-cell suspension, and each kind of cell was made into 3 holes; 1 × 10^5^fibroblasts per well was accurately added to the upper chamber hole, and the cell suspension volume was 100 ~ 200 μL, which was placed in 5%CO_2_ 37°C for 24 h. When the culture time was over, the upper chamber fluid was discarded, carefully removed the upper chamber, and wiped off the cells that had not passed through the membrane with a wet cotton swab. The Transwell plates were fixed with 10% formalin and stained with 0.05% crystal violet for 30 min. The number of cells passing through the membrane was directly observed under an inverted microscope.

### Dual-luciferase reporter assay

Human embryonic kidney cell 293 (HEK293; Procell, Wuhan, China) cells (4.5 × 10^4^) were grown in a 48-well plate and cultured to 70% confluence. Then circPTPN12-WT or circPTPN12-MUT and miR-21-5p mimics or Phage NC (Kosmo Biotechnology, Tianjin, China) were co-transfected into HEK293 cells with Lipofectamine® 2000. After transfection48 h, luciferase activity was verified according to the manufacturer’s instructions as described [30].

### RNA immunoprecipitation (RIP) assay

RIP was carried out as described [31].2 × 10^7^ fibroblasts were collected and lysed with the same volume of H1P lysate. The diameter of centrifugation was 420 mm, and the centrifugal diameter was 10 min. The supernatant was obtained. According to the instructions of RIP kit, the buffer (including RNase inhibitor, protease inhibitor, DNase) and 100 μL cell lysate were added to the EP tube containing magnetic beads, IgG or Ago2 antibody was added, incubated overnight at 4°C, The supernatant was discarded, and 500 μL RIP Wash Buffer was washed for 6 times. 15 μL diethylpyrocarbonate was used to dissolve and purify RNA in water, stored at −80°C. The group without antibody was the positive control group (Input), the group with IgG antibody was the negative control group (anti-lgG), and the group with Ago2 antibody was the experimental group (anti-Ago2).

### RNA pull-down assay

Pull-down experiments were conducted as described [32]. A total of 1 × 10^7^fibroblasts were collected, lysed, and broken by ultrasound. The circPTPN12 probe was incubated with M-280 magnetic beads (Life Technologies) at 25°C for 2 h to form probe-coated beads. The cell lysate containing circPTPN12 probe or oligonucleotide probe was incubated at 4°C overnight. After washing with a washing buffer, the RNA mixture bound to the beads was eluted with RNasy Mini Kit (QIAGEN, Hilden, Germany) for qRT-PCR.

### Western blot

Western blot analysis of protein expression was performed as described previously [33]. The lysate was added to each group of fibroblasts, and the supernatant was absorbed by 4°C. Protein quantification was performed by bicinchoninic acid (BCA) assay. After sodium dodecyl sulfate-polyacrylamide gel electrophoresis (SDS-PAGE) electrophoresis, the membrane was transferred and sealed, and SMAD7, cyclinD1, c-myc, and the internal reference protein GAPDH primary antibody (1: 1000) were added and incubated overnight at 4°C. After TBST washing, the sheep anti-rabbit second antibody (1: 2500) was incubated at room temperature for 1 h. The mixed chemiluminescence solution of the same volume reacted with PVDF membranes (Millipore, Bedford, MA, USA), exposed and preserved. The image analysis was performed using ImageJ (National Institutes of Health, MD, USA) software.

## Statistical analysis

Statistical analysis was carried out by SPSS21.0 statistical software (SPSS Inc., Chicago, IL, USA). The measurement data were expressed as mean ± standard deviation (x ± s). Each independent experiment was repeated three times. The independent sample t-test was used for the comparison between groups.

## Results

This study investigated the potentials of circPTPN12 in keloids and explored the mechanism. In *in vitro* assays, we confirmed that silencing circPTPN12 could accelerate viability and suppress apoptosis of keloid fibroblasts via miR-21-5p/SMAD7 axis.

## CircPTPN12 declined in keloid, and silencing circPTPN12 could promote the growth of keloid fibroblasts

**Figure 1.**
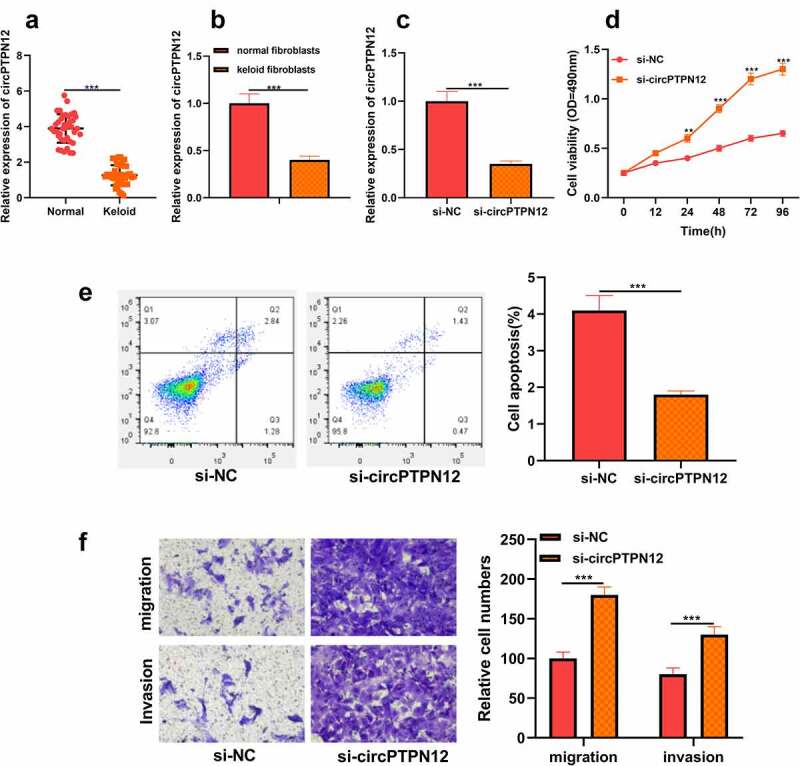
CircPTPN12 declined in keloid tissue and keloid fibroblasts, and silencing circPTPN12 could promote the growth of keloid fibroblasts. (a) qRT-PCR was applied to detect the expression of circPTPN12 in keloid tissue. (b) qRT-PCR was applied to detect the expression of circPTPN12 in keloid fibroblasts. (c) qRT-PCR was used to detect the transfection efficiency of circPTPN12 knockdown. (d) MTT assay was applied to assess the cell viability of keloid fibroblasts. (e) Flow cytometry was used to detect cell apoptosis of keloid fibroblasts. (f) Transwell assay was applied to assess the cell migration and invasion of keloid fibroblasts. ** *P* < 0.01, *** *P* < 0.001.

To discover the role of circPTPN12 in keloid formation, firstly, the level of circPTPN12 in keloid tissue. As shown in Figure 1(a), it was revealed that circPTPN12 was dramatically down-regulated in keloid tissue compared with normal skin tissue. Moreover, it was also implied that the expression of circPTPN12 in keloid fibroblasts was remarkably lower than that in normal fibroblasts (*P* < 0.05, Figure 1(b)). Therefore, we speculate that circPTPN12 may play a significant part in keloid formation. To study the role of circPTPN12 in keloid formation, we used si-circPTPN12 to carry out loss of function. First of all, we evaluated the transfection efficiency of si-circPTPN12 by detecting the expression of circPTPN12. The results illustrated that si-circPTPN12 could markedly attenuate the level of circPTPN12, revealing that si-circPTPN12 can be applied to the subsequent loss of function experiments (*P* < 0.05, Figure 1(c)). MTT and flow cytometry demonstrated that silencing circPTPN12 markedly increased the survival rate of keloid fibroblasts and inhibited the apoptosis rate of keloid fibroblasts (*P* < 0.05, Figure 1(d,e)). Otherwise, the Transwell experiment proved that silencing circPTPN12 also promoted the migration and invasion of keloid fibroblasts(*P* < 0.05, Figure 1(f)). This indicates that circPTPN12 knockout promotes the proliferation, migration, and invasion of keloid fibroblasts and inhibits the apoptosis of keloid fibroblasts, suggesting that circPTPN12 may play a negative part in keloid formation.

## CircPTPN12 could sponge miR-21-5p in keloid fibroblasts

**Figure 2.**
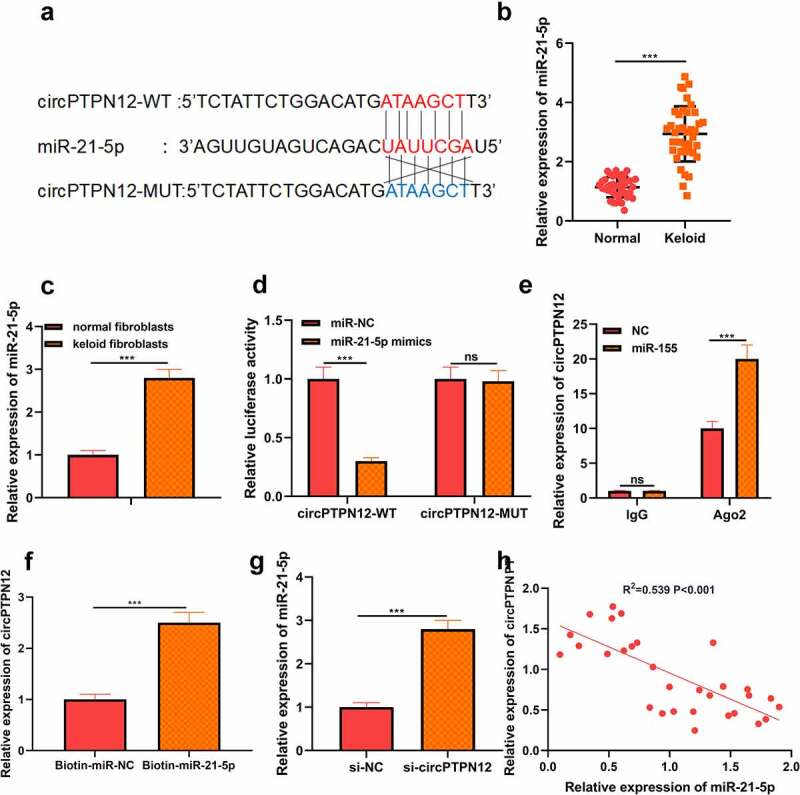
CircPTPN12 could sponge miR-21-5p in keloid fibroblasts. (a) CircPTPN12 contained a conserved binding site of miR-21-5p. (b) qRT-PCR was applied to detect the expression of miR-21-5p in keloid tissue. (c) qRT-PCR was applied to detect the expression of miR-21-5p in keloid fibroblasts. (d) The binding of circPTPN12 and miR-21-5p was verified by dual-luciferase reporter gene assay. (e, f)The combination of circPTPN12 and miR-21-5p was verified by RNA immunoprecipitation and pull-down assays. (g) qRT-PCR was used to detect the level of miR-21-5p after low expression of circPTPN12. (h) Pearson correlation analysis was used to detect the correlation between circPTPN12 and miR-21-5p in keloid tissue. *** *P* < 0.001.

Using the StarBase tools to predict the potential target miRNA of circPTPN12. As shown in Figure 2(a), miR-21-5p was found to have a binding site for circPTPN12. By detecting the expression of miR-21-5p in keloid tissues and fibroblasts, we found that the expression of miR-21-5p in keloid tissues and fibroblasts was higher than that in normal skin tissues and normal fibroblasts, respectively (*P* < 0.05, Figure 2(b,c)). To further confirm the binding relationship, we carried out the dual-luciferase reporter gene experiment. We found that miR-21-5p mimics could dramatically reduce the luciferase activity driven by the circPTPN12-WT reporter vector. In contrast, the luciferase activity of circPTPN12-MUT reporter vector had no effect, which confirmed the complementary binding relationship between circPTPN12 and miR-21-5p (*P* < 0.05, Figure 2(d)). To further identify whether miR-21-5pis can function as a sponge miRNA of circPTPN12. RIP analysis showed that circPTPN12 and miR-21-5p in Ago2 particles were more abundant than those in IgG particles (*P* < 0.05, Figure 2(e)). Furthermore, The RNA pull-down assay with biotinylated miR-21-5p (miR-21-5p-bio) probe increased the level of circPTPN12 compared with the miR-21-5p investigation in the control group (NC-bio) (*P* < 0.05, Figure 2(f)). At the same time, qRT-PCR results indicated that circPTPN12 silencing could accelerate the level of miR-21-5p in keloid fibroblasts (*P* < 0.05, Figure 2(g)). Furthermore, correlation analysis showed a negative correlation between the expression of miR-21-5p and circPTPN12 in keloid tissues (*P* < 0.05, Figure 2(h)). All data supported that miR-21-5p could be a sponge ofcircPTPN12 in keloid.

## MiR-21-5p directly targeted SMAD7 in keloid fibroblasts

**Figure 3.**
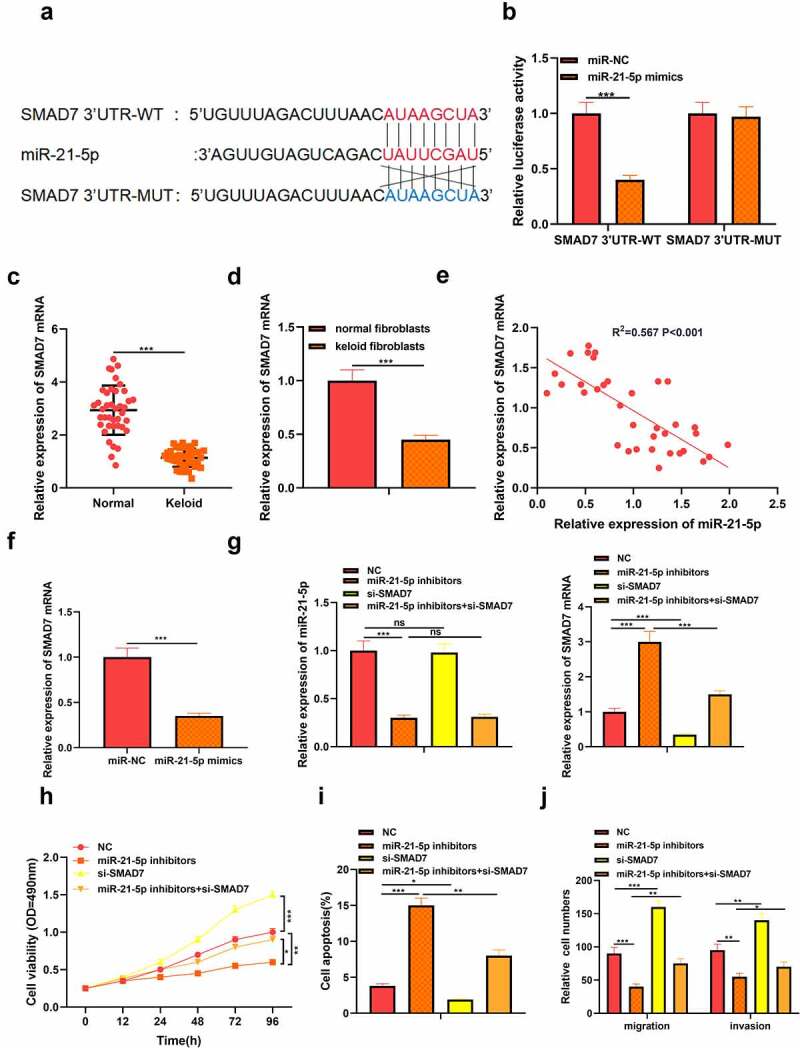
MiR-21-5p directly targeted SMAD7 in keloid fibroblasts. (a) SMAD7 contained a conserved binding site of miR-21-5p. (b) The binding of SMAD7 and miR-21-5p was verified by dual-luciferase reporter gene assay. (c) qRT-PCR was applied to detect the expression of SMAD7 in keloid tissue. (d) qRT-PCR was applied to detect the expression of SMAD7 in keloid fibroblasts. (e) Pearson correlation analysis was used to detect the correlation between SMAD7 and miR-21-5p in keloid tissue. (f) qRT-PCR was used to detect the level of SMAD7 after overexpression of miR-21-5p. Cotransfection of miR-21-5p inhibitors and si-SMAD7 into keloid fibroblasts. (g) The levels of miR-21-5p and SMAD7 were detected by qRT-PCR. (h) MTT assay was applied to assess the cell viability of keloid fibroblasts. (i) Flow cytometry was used to detect cell apoptosis of keloid fibroblasts. (j) Transwell assay was applied to assess the cell migration and invasion of keloid fibroblasts. * *P* < 0.05, ** *P* < 0.01, *** *P* < 0.001.

At the same time, the target gene of miR-21-5p was predicted by StarBasetools. We found complementary binding sites between SMAD7 3’UTR and miR-21-5p (Figure 3(a)). Similarly, dual-luciferase reporter experiments with SMAD7 3’UTR-WT and SMAD7 3’UTR-MUT. The results showed that miR-21-5p mimics memorably inhibited the luciferase activity of SMAD7 3’UTR-WT but did not affect the luciferase activity of SMAD7 3’UTR-MUT (Figure 3(b)). Otherwise, we detected the level of SMAD7 in keloid tissues and fibroblasts by qRT-PCR analysis and found that the expression of SMAD7 in keloid tissue and fibroblasts decreased (*P* < 0.05, Figure 3(c,d)). Pearson’s analysis confirmed a negative correlation between the expression of SMAD7 and the expression of miR-21-5p (*P* < 0.05, Figure 3(e)). In addition, we found that miR-21-5p suppressed the level of SMAD7 in keloid fibroblasts transfected with miR-21-5p mimics (*P* < 0.05, Figure 3(f)). Therefore, we determined that SMAD7 was the target of miR-21-5p in keloid. To further confirm the role of miR-21-5p/SMAD7 in keloid, miR-21-5p inhibitor and SMAD7 low expression plasmid were co-transfected into keloid fibroblasts, and the transfection efficiency was verified by qRT-PCR (*P* < 0.05, Figure 3(g)). MTT assay showed that the survival rate of keloid fibroblasts decreased after miR-21-5p inhibitor transfection while increased after low expression of SMAD7 (*P* < 0.05, Figure 3(h)). Moreover, flow cytometry showed that apoptosis of keloid fibroblasts increased after miR-21-5p inhibitor transfection but decreased after low expression of SMAD7 (*P* < 0.05, Figure 3(i)). Otherwise, the migration and invasion level of keloid fibroblasts decreased after miR-21-5p inhibitor transfection, while the level increased after low expression of SMAD7(*P* < 0.05, Figure 3(j)). This suggested that the miR-21-5p/SMAD7 axis could regulate keloid progression.

## SilencingcircPTPN12 down-regulated SMAD7 to promote keloid formation by targeting miR-21-5p

**Figure 4.**
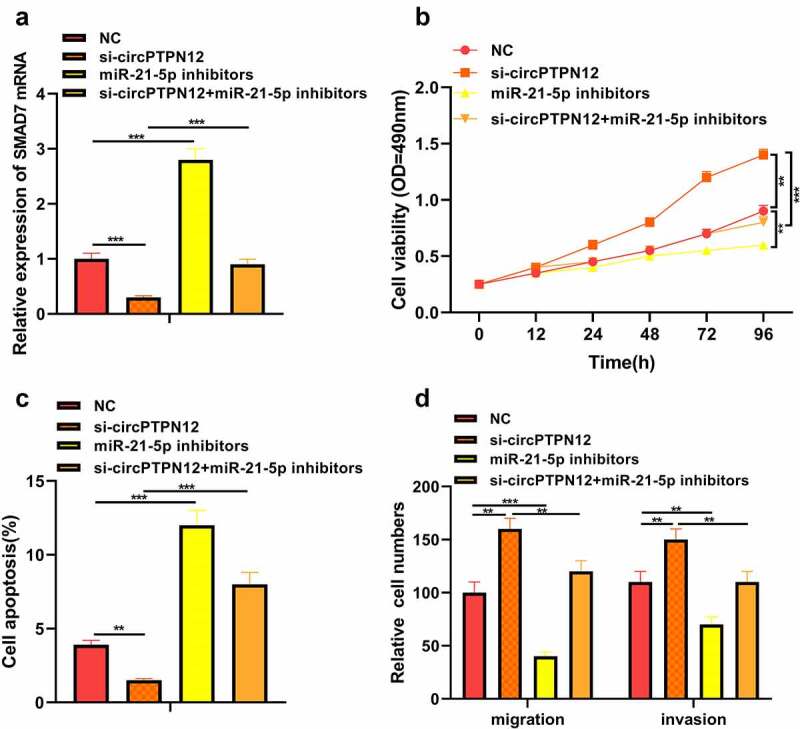
Silencing circPTPN12 targeted miR-21-5p/SMAD7 axis and activated the Wnt pathway to strengthen keloid fibroblasts’ growth. Cotransfection of miR-21-5p inhibitors and si-circPTPN12 into keloid fibroblasts. (a) The level of SMAD7 was detected by qRT-PCR. (b) MTT assay was applied to assess the cell viability of keloid fibroblasts. (c) Flow cytometry was used to detect cell apoptosis of keloid fibroblasts. (d) Transwell assay was applied to assess the cell migration and invasion of keloid fibroblasts. (e) Western blot was applied to assess the levels of cyclinD1 and c-myc. (f) The mechanism diagram indicated that silencing circPTPN12 targets the miR-21-5p/SMAD7 axis and activates the Wnt signal pathway, thus affecting the progression of keloid. ** *P* < 0.01, *** *P* < 0.001.

To further verify whether circPTPN12 regulates keloid formation through the miR-21-5p/SMAD7 axis, we co-transfected si-circPTPN12 and miR-21-5p inhibitors into keloid fibroblasts. The successful transfection of si-circPTPN12 and miR-21-5p inhibitors was confirmed (*P* < 0.05, Figure 4(a)). Subsequently, MTT assay and flow cytometry indicated that miR-21-5p inhibitors could reverse the effect of circPTPN12 gene knockout on proliferation and apoptosis of keloid fibroblasts (*P* < 0.05, Figure 4(b,c)). Otherwise, the increase in the number of migration and invasion keloid fibroblasts promoted by circPTPN12 silencing could be restored by miR-21-5p inhibitors. Thus, we confirmed that miR-21-5p plays a vital role in regulating keloid formation by circPTPN12.

## Silencing circPTPN12 targeted miR-21-5p/SMAD7 axis and activated Wnt pathway to strengthen the growth of keloid fibroblasts

**Figure 5.**
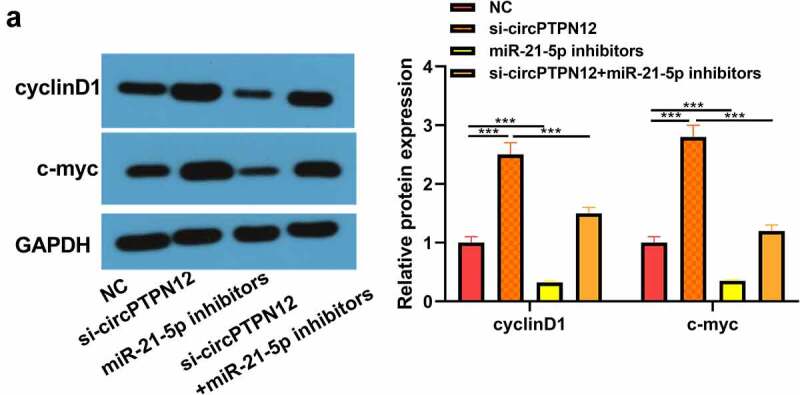
Silencing circPTPN12 targeted miR-21-5p/SMAD7 axis and activated the Wnt pathway to strengthen keloid fibroblasts’ growth. (a) The protein levels of cyclinD1 and c-myc were induced after silencing circPTPN12, while partly reversed by inhibition of miR-21-5p in keloid fibroblasts. Data were shown as mean ± SD.*** *P* < 0.001.

The proteins cyclinD1 and c-myc associated with the Wnt signaling pathway were up-regulated in keloid fibroblasts after low expression of circPTPN12, but down-regulated after down-regulation miR-21-5p (*P* < 0.05, Figure 5(a)). Therefore, the Wnt signaling pathway in keloid might be activated by down-regulation of circPTPN12 and up-regulation of miR-21-5p. Our results indicate that silencing circPTPN12 targets the miR-21-5p/SMAD7 axis and activates the Wnt signaling pathway, thus affecting the progression of keloid.

## Discussion

There are still many problems to be solved in the clinical treatment of keloid, including drug injection, surgical resection, and perioperative radiotherapy, but recurrence and postoperative deformities cannot be avoided entirely [34,35]. Therefore, keloid treatment is changing from simple surgical resection to comprehensive treatment, and combined gene therapy is one of the research hotspots. In contrast, the etiology and molecular pathology of keloid formation and progression are still unclear. Genetic susceptibility is considered to be important in the pathogenesis of keloid formation. However, environmental factors and epigenetic mechanisms may also play a key role. Epigenetic modification is an important research field in studying the molecular pathogenesis of keloid [36,37]. In the present research, we devoted ourselves to the function of circRNAs in keloid. It is found that circPTPN12, a new type of circRNA, may play an inhibitory role in keloid formation.

In recent years, circRNAs have been referred to as the progress of keloid. CircRNAs were differentially expressed in keloid fibroblasts, and circRNAs were mainly referred to as apoptosis, adhesion plaques, PI3K-Akt, and metabolic pathways [6]. Similarly, Zhang et al have detected an average of 120 and 12 circRNAs up-regulated and down-regulated in keloid compared with normal controls by high-throughput sequencing [38]. These findings suggest that circRNAs are essential in keloids. Moreover, circPTPN12 has been shown to promote the progression of endometrial fibrosis [10]. The present study proposed that circPTPN12 was low expressed in keloid, and silencing circPTPN12 induced the viability of keloid fibroblasts and declined apoptosis, which indicated that circPTPN12 had an inhibitory effect on the growth of keloid. Keloid scars are characterized by collagen accumulation in the extracellular matrix (ECM). Mech of the literature on ECM acknowledges the involvement of circRNAs in the degradation or accumulation of ECM. Circ_15698 accelerated ECM accumulation in diabetic nephropathy [39].Circ_4099 regulated ECM synthesis by arresting miR-616-5p to inhibit SOX9 in intervertebral disc degeneration [40]. Based on these, we deduce that circPTPN12 may participate in collagen production and accumulation in ECM, which could be a breakthrough point for future research.

MiRNAs are small non-coding RNAs involved in skin fibrosis. The length of these small RNA ranges from 18 to 25 nucleotides. It modifies gene expression by binding to the target messenger RNA (mRNA), which leads to the degradation of target mRNA or inhibition of translation into proteins [41]. It is reported that many miRNAs are abnormally expressed in the keloid. For instance, miR-196b-5p is declined in keloid. Additionally, miR-196b-5p inhibitors can promote the viability of keloid fibroblasts and the level of extracellular matrix, which is achieved by targeting FGF2 [42]. Jin et al have confirmed that miR-124-3p promotes apoptosis of keloid fibroblasts and inhibits fibroblast-induced angiogenesis by targeting TGFβR1 [43]. Otherwise, Studies have confirmed that circ_101238/miR-138-5p/CDK6 axis plays a potential regulatory role in keloid [8]. Lv and other related reports also show that circCOL5A1plays the role of ceRNA and induces keloid formation by sponging miR-7-5p to recruit Epac1 [38]. Construction of interaction network based on circRNA-miRNA-mRNA [44].Evidence suggests that miR-21-5p affects ECM formation in uterine fibroids [45]. This study assessed the mechanism of circPTPN12 in keloid. Here, it was revealed that circPTPN12 sponged miR-21-5p. Moreover, enhanced miR-21-5p in keloids, which was similar to the results of Yan et al [17]. Furthermore, si-circPTPN12 induced the phenotype of keloid fibroblasts, which was reversed by miR-21-5p inhibitors, showing that miR-21-5p was concerned with regulating keloid formation by circPTPN12. Additionally, SMAD7, as a downstream target of miR-21-5p, was declined in keloids. Furthermore, it was revealed that the low expression of SMAD7 reversed the function of miR-21-5p inhibitors. In addition, the level of SMAD7 was adjusted by circPTPN12/miR-21-5p axis. Consistent with previous reports by Li and others, SMAD7 may be a target for treating keloid [20]. The interaction of circPTPN12/miR-21-5p/SMAD7 axis in keloid can further reveal that circPTPN12 inhibits keloid formation.

Wnt protein is a family of secretory proteins that exist widely in the body. Reports have confirmed that SMAD7 can contribute to pituitary adenomas’ proceeding by activating the Wnt pathway [46]. Furthermore, low expression of miR-17-5p protects nasal epithelial cells from injury by targeting SMAD7 to inactivate the Wnt pathway [47]. In this study, we found that silencing circPTPN12 activates the Wnt signal pathway by regulating the miR-21-5p/SMAD7 axis, thus affecting the progression of keloid. Therefore, this study has some limitations. First of all, this study focuses on the effects of circPTPN12 on the proliferation, migration, invasion, and apoptosis of keloid fibroblasts. Previous studies have confirmed that malignant phenotypes such as extracellular matrix and angiogenesis play an essential role in studying keloids [42,43]. Therefore, the research needs to be further investigated. Otherwise, this study preliminarily explored the role of circPTPN12 in keloid from the cellular level and tissue level, which needs to be further confirmed by animal experiments. Finally, it is not clear whether circPTPN12 regulates the occurrence of keloid by affecting the expression of other downstream proteins or regulating different signaling pathways. Taken together, we believe that silencing circPTPN12 promotes the proliferation, migration, invasion, and inhibition of apoptosis of keloid fibroblasts by targeting miR-21-5p/SMAD7 pathway and activating Wnt signaling pathway, thus promoting the formation of keloid. This study reveals for the first time the mechanism of the effect of circPTPN12 on keloid formation, and the appearance of circPTPN12/miR-21-5p/SMAD7 axis also provides a reliable target for the treatment of keloid.

In summary, circPTPN12 was lowly expressed in keloid, while miR-21-5p was highly expressed, and the correlations were negative. Silencing with circPTPN12 expression could promote fibroblasts’ proliferation and migration ability and inhibit apoptosis, which may act by targeting the negative regulation of miR-21-5p and may become a molecular target for scar therapy. However, this study only investigated the effect of circPTPN12/miR-21-5p axis on the proliferation, migration, and apoptosis of scar cells through *in vitro* cellular assays and circPTPN12/miR-21-5p axis on scar formation needs to be investigated *in vivo* through nude mice experiments.

## Conclusion

This is the first study to report that silencingcirc PTPN12 promotes keloid fibroblasts’ growth to the best of our knowledge. At least in part through the upregulation of miR-21-5p expression and, therefore, the downregulation of SMAD7 expression. The above findings indicate that the circPTPN12/miR-21-5p/SMAD7 axis may be a promising therapeutic target in keloid.

## Data Availability

The data used to support the findings of this study are available from the corresponding author upon request.

## Abbreviation

CircRNA: circular RNA; miRNA: micro RNA; SMAD7: SMAD family member 7; FBS: fetal bovine serum; qRT-PCR: quantitative Real-time PCR; DMSO: dimethyl sulfoxide; RIP: RNA binding protein immunoprecipitation assay; OD: optical density; BCA: bicinchoninic acid; SDS-PAGE: sodium dodecyl sulfate-polyacrylamide gel electrophoresis; ECM: extracellular matrix

## References

[1] Tan S, Khumalo N, Bayat A. Understanding keloid pathobiology from a quasi-neoplastic perspective: less of a scar and more of a chronic inflammatory disease with cancer-like tendencies. Front Immunol. 2019;10:1810. 31440236 10.3389/fimmu.2019.01810 PMC6692789

[2] Ud-Din S, Bayat A. Keloid scarring or disease: unresolved quasi-neoplastic tendencies in the human skin. Wound Repair Regen. 2020;28(3):422–426. 31943508 10.1111/wrr.12793

[3] Lee S, Kim SK, Park H, et al. Contribution of Autophagy-Notch1-mediated NLRP3 inflammasome activation to chronic inflammation and fibrosis in keloid fibroblasts. Int J Mol Sci. 2020;21(21):8050. 10.3390/ijms21218050 PMC7663397 33126764

[4] Ekstein SF, Wyles SP, Moran SL, et al. Keloids: a review of therapeutic management. Int J Dermatol. 2021;60(6):661–671. 32905614 10.1111/ijd.15159 PMC7940466

[5] Bijlard E, Verduijn GM, Harmeling JX, et al. Optimal high-dose-rate brachytherapy fractionation scheme after keloid excision: a retrospective multicenter comparison of recurrence rates and complications. Int J Radiat Oncol Biol Phys. 2018;100(3):679–686. 29249529 10.1016/j.ijrobp.2017.10.044

[6] Zhang Z, Yu K, Liu O, et al. Expression profile and bioinformatics analyses of circular RNAs in keloid and normal dermal fibroblasts. Exp Cell Res. 2020;388(1):111799. 31904383 10.1016/j.yexcr.2019.111799

[7] Lin Z, Long F, Zhao M, et al. The role of circular RNAs in hematological malignancies. Genomics. 2020;112(6):4000–4008. 32634468 10.1016/j.ygeno.2020.06.051

[8] Yang D, Li M, Du N. Effects of the circ_101238/miR-138-5p/CDK6 axis on proliferation and apoptosis keloid fibroblasts. Exp Ther Med. 2020;20(3):1995–2002. 32782509 10.3892/etm.2020.8917 PMC7401192

[9] Wang B, Yin L, Zhang HM, et al. circNRIP1 facilitates keloid progression via FXR1‑mediated upregulation of miR‑503‑3p and miR‑503‑5p. Int J Mol Med. 2021;47(5):70. 33649815 10.3892/ijmm.2021.4903 PMC7952250

[10] Song M, Zhao G, Sun H, et al. circPTPN12/miR-21-5 p/Np63alpha pathway contributes to human endometrial fibrosis. Elife. 2021;10. DOI:10.7554/eLife.65735. PMC8208816 34132637

[11] Zhang ZY, Gao XH, Ma MY, et al. CircRNA_101237 promotes NSCLC progression via the miRNA-490-3p/MAPK1 axis. Sci Rep. 2020;10(1):9024. 32494004 10.1038/s41598-020-65920-2 PMC7270109

[12] Zhang J, Liu Y, Shi G. The circRNA-miRNA-mRNA regulatory network in systemic lupus erythematosus. Clin Rheumatol. 2021;40(1):331–339. 32533339 10.1007/s10067-020-05212-2

[13] Li Q, Li B, Li Q, et al. Exosomal miR-21-5p derived from gastric cancer promotes peritoneal metastasis via mesothelial-to-mesenchymal transition. Cell Death Dis. 2018;9(9):854. 30154401 10.1038/s41419-018-0928-8 PMC6113299

[14] Nasci VL, Chuppa S, Griswold L, et al. miR-21-5p regulates mitochondrial respiration and lipid content in H9C2 cells. Am J Physiol Heart Circ Physiol. 2019;316(3):H710–H721. 30657727 10.1152/ajpheart.00538.2017 PMC6459316

[15] Zhang X, Li X, Li B, et al. miR-21-5p protects hippocampal neurons of epileptic rats via inhibiting STAT3 expression. Adv Clin Exp Med. 2020;29(7):793–801. 32745381 10.17219/acem/121929

[16] Yan L, Wang LZ, Xiao R, et al. Inhibition of microRNA-21-5p reduces keloid fibroblast autophagy and migration by targeting PTEN after electron beam irradiation. Lab Invest. 2020;100(3):387–399. 31558773 10.1038/s41374-019-0323-9

[17] Yan L, Cao R, Liu Y, et al. MiR-21-5p links epithelial-mesenchymal transition phenotype with stem-like cell signatures via AKT Signaling in keloid keratinocytes. Sci Rep. 2016;6:28281. 27596120 10.1038/srep28281 PMC5011940

[18] Zhou J, Qie S, Fang H, et al. MiR-487a-3p suppresses the malignant development of pancreatic cancer by targeting SMAD7. Exp Mol Pathol. 2020;116:104489. 32622014 10.1016/j.yexmp.2020.104489

[19] Kleerekooper I, Herbert MK, Kuiperij HB, et al. CSF levels of glutamine synthetase and GFAP to explore astrocytic damage in seronegative NMOSD. J Neurol Neurosurg Psychiatry. 2020;91(6):605–611. 32217788 10.1136/jnnp-2019-322286

[20] Li Q, Fang L, Chen J, et al. Exosomale microRNA-21 Promotes Keloid Fibroblast Proliferation and Collagen Production by inhibiting Smad7. J Burn Care Res. 2021;42(6):1266–1274. 34146092 10.1093/jbcr/irab116

[21] Li X, Wu X. MiR-21-5p promotes the progression of non-small-cell lung cancer by regulating the expression of SMAD7. Onco Targets Ther. 2018;11:8445–8454. 30568467 10.2147/OTT.S172393 PMC6276624

[22] Zhang J, Liu N, Wu X, et al. Identification of differentially expressed circular RNAs in keloid and normal skin tissue by high-throughput sequencing. Dermatol Ther. 2021;34(2):e14745. 33405341 10.1111/dth.14745

[23] Odorisio T, Di Salvio M, Orecchia A, et al. Monozygotic twins discordant for recessive dystrophic epidermolysis bullosa phenotype highlight the role of TGF-beta signalling in modifying disease severity. Hum Mol Genet. 2014;23(15):3907–3922. 24599399 10.1093/hmg/ddu102

[24] Frost JR, Mendez M, Soriano AM, et al. Adenovirus 5 E1A-Mediated suppression of p53 via FUBP1. J Virol. 2018;92(14). DOI:10.1128/JVI.00439-18 PMC6026727 29743362

[25] Shao Y, Song Y, Xu S, et al. Expression profile of circular RNAs in oral squamous cell carcinoma. Front Oncol. 2020;10:533616. 33330021 10.3389/fonc.2020.533616 PMC7729060

[26] Zambon AC, Zhang L, Minovitsky S, et al. Gene expression patterns define key transcriptional events in cell-cycle regulation by cAMP and protein kinase A. Proc Natl Acad Sci U S A. 2005;102(24):8561–8566. 15939874 10.1073/pnas.0503363102 PMC1150853

[27] Jiao Y, Wang X, Zhang J, et al. Inhibiting function of human fetal dermal mesenchymal stem cells on bioactivities of keloid fibroblasts. Stem Cell Res Ther. 2017;8(1):170. 28720118 10.1186/s13287-017-0624-0 PMC5516368

[28] Han W, Xu Y, Feng X, et al. NQO-induced DNA-less cell formation is associated with chromatin protein degradation and dependent on A0A1-ATPase in sulfolobus. Front Microbiol. 2017;8:1480. 28855893 10.3389/fmicb.2017.01480 PMC5557786

[29] Wei Y, Zhang F, Zhang T, et al. LDLRAD2 overexpression predicts poor prognosis and promotes metastasis by activating Wnt/beta-catenin/EMT signaling cascade in gastric cancer. Aging (Albany NY). 2019;11(20):8951–8968. 31649207 10.18632/aging.102359 PMC6834412

[30] Sun Y, Qin B. Long noncoding RNA MALAT1 regulates HDAC4-mediated proliferation and apoptosis via decoying of miR-140-5p in osteosarcoma cells. Cancer Med. 2018;7(9):4584–4597. 30094957 10.1002/cam4.1677 PMC6144160

[31] Sun Y, Yang Z, Zheng B, et al. A novel regulatory mechanism of smooth muscle alpha-actin expression by NRG-1/circACTA2/miR-548f-5p axis. Circ Res. 2017;121(6):628–635. 28698179 10.1161/CIRCRESAHA.117.311441

[32] Garcia-Dominguez M, March-Diaz R, Reyes JC. The PHD domain of plant PIAS proteins mediates sumoylation of bromodomain GTE proteins. J Biol Chem. 2008;283(31):21469–21477. 18502747 10.1074/jbc.M708176200

[33] Lee KJ, Kim HA, Kim PH, et al. Ox-LDL suppresses PMA-induced MMP-9 expression and activity through CD36-mediated activation of PPAR-g. Exp Mol Med. 2004;36(6):534–544. 15665586 10.1038/emm.2004.68

[34] Lee HJ, Jang YJ. Recent understandings of biology, prophylaxis and treatment strategies for hypertrophic scars and keloids. Int J Mol Sci. 2018;19(3):711. 10.3390/ijms19030711 PMC5877572 29498630

[35] Berman B, Maderal A, Raphael B. Keloids and hypertrophic scars: pathophysiology, classification, and treatment. Dermatol Surg. 2017;43(Suppl 1):S3–S18. 27347634 10.1097/DSS.0000000000000819

[36] Naik PP. Review on novel targets and therapies for keloids. Clin Exp Dermatol. 2021. DOI:10.1111/ced.14920 34480483

[37] Stevenson AW, Deng Z, Allahham A, et al. The epigenetics of keloids. Exp Dermatol. 2021;30(8):1099–1114. 34152651 10.1111/exd.14414

[38] Lv W, Liu S, Zhang Q, et al. Circular RNA CircCOL5A1 sponges the MiR-7-5p/Epac1 axis to promote the progression of keloids through regulating PI3K/Akt signaling pathway. Front Cell Dev Biol. 2021;9:626027. 33553184 10.3389/fcell.2021.626027 PMC7859531

[39] Wei C, Zhang R, Cai Q, et al. MicroRNA-330-3p promotes brain metastasis and epithelial-mesenchymal transition via GRIA3 in non-small cell lung cancer. Aging (Albany NY). 2019;11(17):6734–6761. 31498117 10.18632/aging.102201 PMC6756898

[40] Wang H, He P, Pan H, et al. Circular RNA circ-4099 is induced by TNF-alpha and regulates ECM synthesis by blocking miR-616-5p inhibition of Sox9 in intervertebral disc degeneration. Exp Mol Med. 2018;50(4):1–14. 10.1038/s12276-018-0056-7 PMC5938034 29651107

[41] Wang D, Xin L, Lin JH, et al. Identifying miRNA-mRNA regulation network of chronic pancreatitis based on the significant functional expression. Medicine (Baltimore). 2017;96(21):e6668. 28538367 10.1097/MD.0000000000006668 PMC5457847

[42] Yang J, Deng P, Qi Y, et al. NEAT1 knockdown inhibits keloid fibroblast progression by miR-196b-5p/FGF2 axis. J Surg Res. 2021;259:261–270. 33162101 10.1016/j.jss.2020.09.038

[43] Jin J, Jia Z, Luo X, et al. Long non-coding RNA HOXA11-AS accelerates the progression of keloid formation via miR-124-3p/TGFβR1 axis. Cell Cycle. 2020;19(2):218–232. 31878829 10.1080/15384101.2019.1706921 PMC6961662

[44] Xiong DD, Dang YW, Lin P, et al. A circRNA-miRNA-mRNA network identification for exploring underlying pathogenesis and therapy strategy of hepatocellular carcinoma. J Transl Med. 2018;16(1):220. 30092792 10.1186/s12967-018-1593-5 PMC6085698

[45] Cardozo ER, Foster R, Karmon AE, et al. MicroRNA 21a-5p overexpression impacts mediators of extracellular matrix formation in uterine leiomyoma. Reprod Biol Endocrinol. 2018;16(1):46. 29747655 10.1186/s12958-018-0364-8 PMC5946472

[46] Shen DW, Li YL, Hou YJ, et al. MicroRNA-543 promotes cell invasion and impedes apoptosis in pituitary adenoma via activating the Wnt/beta-catenin pathway by negative regulation of Smad7. Biosci Biotechnol Biochem. 2019;83(6):1035–1044. 30973065 10.1080/09168451.2019.1591260

[47] Huang N, Li W, Wang X, et al. MicroRNA-17-5p aggravates lipopolysaccharide-induced injury in nasal epithelial cells by targeting Smad7. BMC Cell Biol. 2018;19(1):1. 29433423 10.1186/s12860-018-0152-5 PMC5809994

